# A physics-based model of swarming jellyfish

**DOI:** 10.1371/journal.pone.0288378

**Published:** 2023-07-10

**Authors:** Erik Gengel, Zafrir Kuplik, Dror Angel, Eyal Heifetz

**Affiliations:** 1 Department of Geophysics, Porter School of the Environment and Earth Sciences, Tel Aviv University, Tel Aviv, Israel; 2 The Steinhardt Museum of Natural History, Tel Aviv University, Tel Aviv, Israel; 3 The Leon Recanati Institute for Maritime Studies, University of Haifa, Mount Carmel, Haifa, Israel; Sao Paulo State University Julio de Mesquita Filho Bauru Campus Faculty of Sciences: Universidade Estadual Paulista Julio de Mesquita Filho Faculdade de Ciencias Campus de Bauru, BRAZIL

## Abstract

We propose a model for the structure formation of jellyfish swimming based on active Brownian particles. We address the phenomena of counter-current swimming, avoidance of turbulent flow regions and foraging. We motivate corresponding mechanisms from observations of jellyfish swarming reported in the literature and incorporate them into the generic modelling framework. The model characteristics is tested in three paradigmatic flow environments.

## Introduction

Scyphozoans populate the oceans since the late proterozoic eon and according to geological records they are one of the first multicellular organisms on planet Earth [1]. The life cycle of scyphozoans is complex including in many species the tiny, cryptic, benthic stage of the polyp. But, most prominent is the planktonic stage of the medusa, better known as *jellyfish* [2, 3]. Despite their appearance, medusae are among the most efficient swimmers in the oceans [4, 5] and their massive occurrence in different habitats has major impacts on the biosphere, tourism, and economics [6–9]. While jellyfish have existed throughout human civilization, the adverse effects of climate change on the ocean habitats are hypothesized to intensify jellyfish blooms and the associated impacts [6, 10].

The fact that scyphomedusa establish swarms and interact with humans on numerous levels has motivated researchers to investigate the relationships between large jellyfish aggregations and environmental conditions [11–16]. Results of these studies have mainly contributed to empiric models for prediction of jellyfish proliferation in estuaries and bays [17–19] or along open coasts [20–22]. These approaches represent the coarse end of a spatio-temporal continuum of models, ranging from regional scale to the level of single-agent swimming. Indeed, many studies have examined the efficiency of different propulsion mechanisms in single medusae, experimentally [23–25] and computationally [26–33], based on detailed observations and direct numerical simulations of bell oscillation and the resulting vortex dynamics in the surrounding water.

In addition, jellyfish respond to a variety of environmental stimuli, affecting the emergence and movement of swarms as a whole. Observations suggest that jellyfish modify their swimming behavior with respect to: the location of other individuals [34], the water temperature [13, 18], salinity [11, 14], advection by water currents, turbulence [19, 23] and the presence of prey [35, 36]. However, there is a large mismatch, between what is known regarding jellyfish ecology and the accuracy of jellyfish swarm prediction based on circulation and distribution models. Thus, there is a great need for a theoretical modelling framework that ties together single-agent dynamics, response mechanisms and swarm behavior, applicable in large scale circulation models [3, 37–40].

Thus, in this paper, we pursue two research goals: First, we propose a mechanistic model framework for the swarming dynamics of jellyfish based on active Brownian particles (ABPs) [41, 42]. A key aspect of the model is that it reaches far beyond existing approaches for modelling of jellyfish proliferation (see Table 1 below): Jellyfish in the model are considered to move actively and they can make decisions based on environmental information. In particular, we pursue the idea that the environmental stimuli are processed by the neuronal network of a jellyfish. This network generates a coherent strong pulsation rhythm which gives rise to the bell movement in many jellyfish species. Accordingly, we consider a jellyfish in our model to be a moving nonlinear oscillator, or *swarmalators* [43–45]. A swarmalator is the next step towards modelling of agent-based swarm phenomena, combining active-matter research and the theory of nonlinear oscillators [46–49]. In particular the latter allows us to incorporate several important biological features into the model, for example the species-dependent response to external stimuli.

**Table 1:** Comparison of the swarming model Eqs (3)–(11) and Eqs (13)–(16) to previous simulation approaches.

	Model	Jellyfish	Active matter
Type	ABPs Swarmalators [43]	Passive tracers [21, 50] Average models [17, 18] Empiric models [51] Single-agent models [26]	ABPs [41] Continuum models [52, 53]
Dynamic variables	Position **x**_*j*_ Orientation *θ*_*j*_ Phase *φ*_*j*_ Activity $A_{j}$	Position Concentration Jellyfish geometry [27]	Position, velocity Density [54] Polarization [55]
Interactions	Parameterized	–	Parameterized [56] Hydrodynamic [57]
Forcings	Flow **U**_*j*_ Vorticity $C_{j}$ Prey *F*_*j*_	Flow **U**_*j*_ [19, 21] Temperature [18]	Flow **U**_*j*_ Temperature, light chemicals [58]
Parameters	Dynamic in response to environment	Constant	Constant or forced [59, 60]
Scales	1 s—1 h 10 cm—10 m	1 s—1 month 10 cm—100 km	1 ms—1 h 1 nm—1 km
Application	Understanding the interaction of jellyfish in tanks (Local predictions) (Upscaling)	Prediction of blooms in: Estuaries, lagoons, bays [17, 18] Beaching events [12, 21] Hydrodynamics of swimming [24, 32]	Pattern formation in swarms of: Birds, fish, locust, fireflies [61, 62] Pedestrians [63] Bacteria [64]

Comparison between the proposed model, existing models for jellyfish and active matter models. In case of active matter, a focus is mostly put on biological applications. Scales represent rough estimates and refer to the mentioned studies. For the proposed model, we mention further steps in the development in brackets.

The second and main research goal is to adapt the introduced parameters of the model according to the existing literature and experience from field observations [23, 35, 65]. On the one hand, the particle-based description allows to extract a consistent mathematical description of individual behavior by means of data analysis techniques for oscillatory systems [66–71]. On the other hand, the ensemble dynamics can be tested in a transient development process of prediction and continuous parameter adjustment, using physics-informed machine learning [51, 72–74] where the parameter set of this work serves as a starting point. Here we integrated data collected for species of the orders Rhizostomeae and Semaeostomeae, species which are known for their swarming behavior. Despite the differences in the bell shape (prolate vs oblate) and swimming patterns (jet-propulsion vs rowing-propulsion) in adult medusae of the two orders, we have managed to provide an overarching set of parameters. However, given the fact that the fields of active matter dynamics and jellyfish ecology have been largely disconnected up to now, a certain amount of intuition is needed to construct an overarching model and to design numerical tests for its validation. As a consequence, we exploit only a minimal set of mechanisms, related to environmental inputs, while we leave out most of the agent-agent interactions. Nevertheless, the resulting active oscillatory swimmers are able to mimic biological phenomena found in swimming jellyfish [75]. Our analysis and characterization of the model performance focuses on theoretical and computational strategies to investigate pattern formation in networks of moving oscillators from the perspective of active-matter research [52, 55, 56, 76–81] and tries to relate these outcomes to the biological background. We elaborate in the concluion section which theoretic quantities should be extracted from experimental data to strengthen the observational evidence for active-jellyfish modelling.

## Methods

We obtain the flow field *U*(**x**, *t*) (hereafter **x** denotes position on the horizontal plane and *t* denotes time) from the incompressible Navier-Stokes equations which we simulate by means of a second-order in time and space method on a staggered grid using a Successive Over-Relaxation (SOR) pressure-solver [82, 83]. The dynamics of the prey concentration *F*(**x**, *t*) is modelled by an advection-diffusion equation coupled to the flow. We fix the diffusion coefficient of the prey to be *D*_*F*_ = 0.001 m^2^ s^−1^ [84].

The trajectories of the jellyfish are simulated by the second-order stochastic Heun-method [85]. We couple the dynamics of the jellyfish to the flow by using a bi-linear interpolation of the local field quantities onto the position of each single jellyfish agent *j* [86] so that for instance *F*_*j*_ = *F*(**x**_*j*_, *t*).

For the statistical analysis of the swarming behavior we make use of ensemble averages, the Pearson correlation [87] and the indication number:

$$\begin{array}{cc} {\bar{W}}_{M} & =\frac{1}{M}\sum_{j=0}^{M-1}W_{j},\\ {\text{Corr}}_{W^{1},W^{2}} & =({\bar{W^{1}W^{2}}}_{N}-{\bar{W^{1}}}_{N}\;{\bar{W^{2}}}_{N})/\left(\sigma^{1}\sigma^{2}\right),\\ N(W) & ={\bar{H(W-\hat{W})}}_{N}\;. \end{array}$$ (1)

Here *j* denotes the serial number of a jellyfish agent and *M* ≤ *N* is the overall number of agents considered for averaging. *W* can be any simulated field variable, e.g. the concentration of prey. *N* is the total number of agents. In reality jellyfish swarms are composed of hundreds of thousands of individuals in the open sea, or just several few individuals in controlled tank experiments [12, 25, 88, 89]. In this work we use *N* = 128 agents as a tradeoff between theoretical demands and experimental limitations.

To obtain statistically significant results, we average values of Eq (1) over *K* = 16 model runs using a Gaussian kernel with a window of 15 seconds [90]. The resulting double average is denoted as $\langle W\rangle \equiv {\langle {\bar{W}}_{M}\rangle }_{K}$. *σ*^1^ and *σ*^2^ are the standard deviations of variables *W*^1^ and *W*^2^. N(W) measures the fraction of jellyfish that can be found in domains fulfilling the condition $W>\hat{W}$. To compare the performances of active jellyfish and passive tracers, we use the ensemble average for passive tracers $\hat{W}\equiv {\bar{W}}_{\text{passive},N}$ if not stated otherwise. For example regarding prey searching, we expect that jellyfish will try to maximize their access to ambient prey while passive tracers will not show any response. Thus, when for a majority of jellyfish $F_{j}>\hat{F}$ holds, N(F)≈1 indicates an excess of preying performance and most jellyfish are situated in regions where the prey concentration is at least higher than $\hat{F}$. An exemplary separation of the flow domain is depicted in Fig 6. In technical terms, the separation is represented by the Heaviside function H which returns unity if $W>\hat{W}$ and zero otherwise.

### Towards a description of active swarming jellyfish

We model a single jellyfish agent as an active over-damped particle at horizontal position **x** = (*x*, *y*)^⊤^ and time *t*, having a velocity **v**(**x**, *t*) and orientation *θ*(**x**, *t*) [41, 42]. In the following, we propose a dynamic model for the position and the orientation of the agent which allows us to capture paradigmatic behaviors of jellyfish.

Jellyfish process environmental information by a neuronal network of several thousand neurons. The dynamics of this network can be regarded as a perturbed internal dynamics [91–94] in which neurons fire coherently and act as a single large oscillator to drive the bell pulsation. Moreover, it has been shown that the neuronal network of jellyfish features properties of a circadian oscillator [89, 95]. Thus, the bell pulsation can be regarded as the resulting average network oscillation [5, 23, 46]. For such a process it has been shown theoretically [46, 96–101] and empirically [102, 103] that a low-dimensional description, in terms of a phase variable *φ*(*t*) is possible and beneficial for a swift but reliable model development. Moreover, phase dynamics models are computationally light, but at the same time a reliable simplification, as they are based on observational evidence and a fit to the theory [66, 69, 99, 103].

Another aspect of jellyfish motion is that swimming patterns can change abruptly in response to stimuli [65, 75]. In such cases jellyfish will switch from a state of relative inactivity into a state where the frequency and the strength of the bell oscillation increase and the swimming trajectories encompass a significantly larger volume of fluid [36, 104, 105]. Such changes in swimming patterns can even cause a swarm to cross flow barriers [35]. Following such periods of elevated activity, jellyfish will return to a state of relative quiescence. To capture this behavior, we introduce an additional activity variable A which causes parametric switching [101, 106, 107]. In accordance with theoretical studies on relaxation oscillators, this variable can be regarded as the leading-order amplitude perturbation from a limit cycle [98, 108, 109]. We assume that this degree of freedom in the jellyfish dynamics is stimulated by external inputs and that it decays exponentially at a rate $\lambda_{A}$, in order to ensure it returns to a quiescent state.

Following these generic considerations, the dynamics of a single jellyfish *j* in a swarm of *N* agents is described by the four differential equations:

$$\begin{array}{cc} {\dot{x}}_{j} & =v_{j}\left(X,\theta ,\varphi ,A_{j},F_{j},U_{j},t\right)\\ {\dot{\theta }}_{j} & =G_{j}(X,\theta ,A_{j},F_{j}{,|C|}_{j},t)\\ {\dot{\varphi }}_{j} & =\omega_{j}\left(A_{j}\right)+H_{j}\left(X,\theta ,\varphi ,A_{j}\right)\\ {\dot{A}}_{j} & =-\lambda_{A}A_{j}+I_{j}\left(\theta ,F_{j}\right)\;. \end{array}$$ (2)

**Fig 1:**
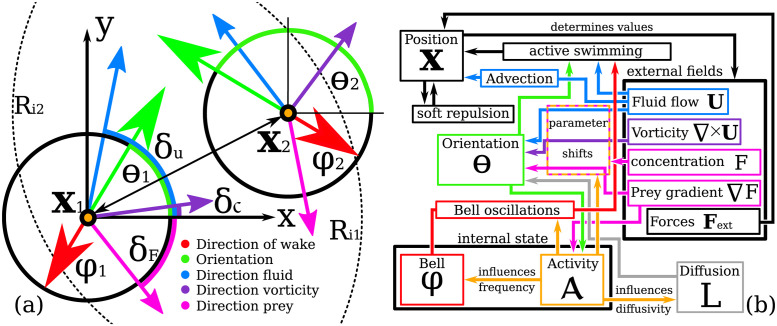
Scheme of the jellyfish agent model. Panel (a): A pair of two agents. Dashed lines indicate the interaction radius *R*_*i*_, The arrow connecting both agents indicates the soft-core repulsion. Colored arrows indicate different orientations according to panel (b). Panel (b): Flow diagram of the state variables **x**, *φ*, *θ* and activity A. Included model mechanisms are advection, swimming against the flow, soft-core repulsion, avoidance of walls, bell oscillations, avoidance of turbulence, local random motion and behavioral shifts due to activity changes (presence of prey). External drivers of the model are the fluid velocity **U**_*j*_, the absolute vorticity ${\left|C\right|}_{j}$ the gradient of prey ∇*F*_*j*_ and the prey concentration *F*_*j*_.

Here, we have used the ensemble notation **X** = [**x**_1_, …, **x**_*N*_], ***φ*** = [*φ*_1_, …, *φ*_*N*_], **θ** = [***θ***_1_, …, *θ*_*N*_]. *F* indicates the prey concentration and **U** denotes the water horizontal current vector. C=∇×U is the vorticity of the water flow. We take its absolute value as a simplified measure for the amount of turbulence in the flow [110]. In the following we exemplify the generic set of Eq (2). For convenience, a scheme of this jellyfish agent model is illustrated in Fig 1. A simplistic comparison of the model to already existing research on active matter and jellyfish dynamics is presented in Table 1. Next we explain the bottom-up rational of the model.

#### Agent activity A

Here we take into account only the presence of prey as an incentive to activate the jellyfish dynamics and assume that the activity evolves according to:

$$\begin{array}{c} {\dot{A}}_{j}=-\lambda_{A}A_{j}+I_{j}\left(\theta ,F_{j}\right)=-\lambda_{A}A_{j}+\left|\nabla F_{j}\right|\frac{1}{2}[|\text{cos}\left(\theta_{j}-\delta_{F}\right)|+\text{cos}\left(\theta_{j}-\delta_{F}\right)]\;. \end{array}$$ (3)

The decay parameter $\lambda_{A}$ corresponds to the time for which an agent is active. We fix this value to $\lambda_{A}=0.005$ s^−1^, equivalent to a characteristic activity time of roughly 3 minutes [65, 111]. We assume that the excitation is a linear function of the magnitude of the local prey concentration gradient |∇*F*_*j*_|. Additionally, the strength of the linear forcing is modulated by the relative orientation of an agent with respect to the direction of the prey concentration gradient. Defining the angle of that gradient as *δ*_*F*_ (satisfying tan(*δ*_*F*_) = ∂_*y*_*F*/∂_*x*_*F*), the term in the square bracket of Eq (3) ensures that only swimming towards the prey (|*θ*_*j*_ − *δ*_*F*_| < *π*/2) evokes an increase in activity. We choose this approach to mimic a basic type of energy saving strategy, by favoring directions towards prey over others.

#### Bell oscillation *φ*

The oscillatory movement of the bell is modelled by a phase oscillator [44, 45, 48, 60, 112]

$$\begin{array}{c} {\dot{\varphi }}_{j}=\omega_{j}\left(A_{j}\right)+H_{j}\left(X,\theta ,\varphi ,A_{j}\right) \end{array}$$ (4)

with a frequency $\omega_{j}\left(A_{j}\right)$. Additionally, the oscillator reacts to perturbations comming from other jellyfish according to a phase coupling function *H*_*j*_(.).

We allow the natural frequency of the bell oscillations to vary according to the activity level A. In order to mimic this coupling, we use the response function:

$$\begin{array}{c} R\left(a,b,S\right)=\frac{aS}{b+S}\;. \end{array}$$ (5)

Here, *a* and *b* are the generic parameters of the response to be set later, and *S* is a stimulus that evokes the response. Generally, R(.) resembles a nonlinear activation function, commonly associated with dynamics in large neuronal networks and employed in machine learning [113]. The reason for this widespread use is that R(.) mimics a quasi-linear response at small stimuli ($R∼\frac{a}{b}S$) and a saturation behavior at large stimuli (R∼a), typical for physiological limitations in biological systems (see Fig 3 panel (a)). The resulting natural frequency response is defined as:

$$\begin{array}{c} \;\omega_{j}=\omega_{j,0}\left[1+R\left(f_{\varphi },f_{\varphi },A_{j}\right)\right]\;, \end{array}$$ (6)

where *ω*_*j*,0_ is the natural frequency of an agent in an inactive state. In this study we fix *ω*_*j*,0_ = *ω*_0_ = 1.2 rad s^−1^ [23, 111] for all agents. Since the bell oscillation frequency in several species can depend on the size of jellyfish, future experimental work is needed to determine the evolution of its distribution for different species during different seasons [50]. Moreover, we seek to allow for a maximal physiological frequency response which we expect to observe when jellyfish escapes its predators. Thus, we fix the frequency response parameter to *f*_*φ*_ = 0.75 [114]. We use a single type of response function, Eq (5), throughout this study both, due to the lack of experimental data and in order to simplify our model. We expect however, that based on future observations, a variety of response functions will be required, similar to paradigmatic models of computational neuroscience [106, 107].

The bell oscillation is subject to multiple external stimuli, foremost the turbulence of the surrounding water due to the bell strokes of other agents. The resulting perturbations exert stresses on the bell tissue and generally impact the oscillatory states of the bell muscles and with these, the neuronal network, causing a potential shortening or lengthening of the bell oscillation period. This variability in periodicity is captured by the phase coupling function *H*_*j*_(.) in Eq (2) [46, 115]. Due to the lack of observations and for the sake of simplicity we set *H*_*j*_(.)≡0, assuming no direct response of the bell to the presence of other agents. Future experiments have to be designed to check for such phase couplings and to extract any potential phase coupling from data [66]. In Eq (2), we indicate the intricate nature of coupling by dependencies on **X** and ***θ*** as the strength of coupling will depend on the position of individuals, the pairwise distance and their orientations.

#### Orientation dynamics *θ*

Jellyfish tend to orient themselves according to different stimuli but they are also subject to the eddy motion induced by their own swimming. The resulting turbulent eddies cause a certain angular drift *L* which acts to reorient the jellyfish, so that

$$\begin{array}{c} {\dot{\theta }}_{j}=G_{j}(X,\theta ,A_{j},F_{j}{,|C|}_{j},t)=L_{j}\left(t\right)+G_{j}(X,\theta ,A_{j},F_{j}{,|C|}_{j})\;. \end{array}$$ (7)

*G*_*j*_(.) is the angular coupling function. The angular diffusion according to *L*_*j*_ is a frequently observed phenomenon on the microscopic [116, 117] and even mesoscopic [118] scale. Although the properties of this diffusion have not been investigated yet for jellyfish, we expect a stochastic approximation of local eddies to be valid. This is because the reorientation is realized by eddies that remain, at least temporarily, attached to a jellyfish [25, 26] before they separate and may lead to communication among different individuals.

Here, we model the phenomenon by a colored noise [119] which allows us to incorporate a simplistic version of correlations and inertial effects of rotation:

$$\begin{array}{c} {\dot{L}}_{j}=-\lambda_{\theta }L_{j}+\eta_{j}\left(t\right),\;\left\langle \eta_{j}\right\rangle =0,\;\left\langle \eta_{j}\eta_{j}^{\prime }\right\rangle =2D\left(A_{j}\right)\delta \left(t-t^{\prime }\right)\;. \end{array}$$ (8)

The parameters λ_*θ*_ is a measure for the correlation time of the noise and *D* is the strength of the white noise *η*_*j*_(*t*). Both, λ_*θ*_ and *D* determine the strength of the angular diffusion. We assume that the resulting motion becomes more prominent when jellyfish search for prey, as individuals start to shuffle water towards their oral arms for feeding purposes [105, 114, 120]. Thus, we allow for an increase of the noise intensity similar to the natural frequencies of agents:

$$\begin{array}{c} D\left(A_{j}\right)=D_{0}\left[1+R\left(f_{\theta },f_{\theta },A_{j}\right)\right]\;. \end{array}$$ (9)

For simplicity we assume equal response parameters *f*_*θ*_ = *f*_*φ*_ and we fix the essentially free parameter *D*_0_ = 0.1rad^2^ s^−3^, which determines the angular diffusion in an inactive state (A=0).

The angular coupling function *G*(.) acts like a potential in the noisy dynamics and causes certain directions to be more favorable than others. In this study, we consider three paradigmatic orientational inputs [75]: First, the agents tend to orient themselves such that they counteract the flow in which they are immersed. Whereas the physiological causes of this behavior are still hypothesized, it is a matter of fact that swimming against the currents increases the survival rate [19]. For example, currents may transport jellyfish to undesired regions such as shore lines and swimming against the flow also increases the chances of catching prey. Second, jellyfish try to avoid regions of turbulent flow in order to optimize their swimming performances and to escape shear flows that may harm their fragile body. Third, jellyfish orient towards the prey concentration, in particular when under starvation [35]. In order to model the interplay of these behaviors, we introduce three separate angular coupling functions $g_{j}\left(\theta_{j}-\delta_{\left(U,C,F\right)}\right)$ with respect to the three local directions $\delta_{\left(U,C,F\right)}$, of current, absolute vorticity gradient and prey:

$$\begin{array}{c} \text{tan}(\delta_{U})=\frac{U_{y}}{U_{x}},\;\text{tan}(\delta_{\left|C\right|})=\frac{\partial_{y}\left|C\right|}{\partial_{x}\left|C\right|},\;\text{tan}(\delta_{F})=\frac{\partial_{y}F}{\partial_{x}F}\;. \end{array}$$ (10)

We assume that the respective angular coupling functions possess a single dominant maximum that can be well approximated by a sinusoidal harmonics [97, 112, 115, 121], resulting in the overall angular dynamics:

$$\begin{array}{c} {\dot{\theta }}_{j}=L_{j}+\varepsilon_{U}\text{sin}(\theta_{j}-\delta_{U})+\varepsilon_{C}\text{sin}(\theta_{j}-\delta_{C})-\varepsilon_{F}\text{sin}(\theta_{j}-\delta_{F})\;. \end{array}$$ (11)

Constructed in this way (for $\varepsilon_{\left(U,C,F\right)}>0)$, the interplay of external orientation inputs tries to establish a stable orientation in parallel of the tumbling.

**Fig 2:**
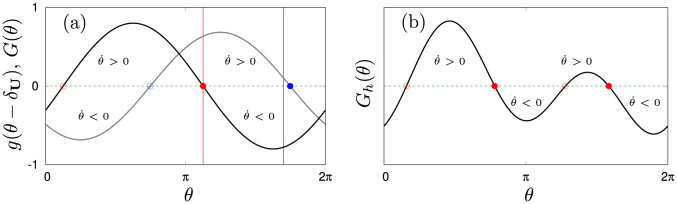
Depicted are examples of the angular dynamics Eq (7) in the absence of noise (*L*_*j*_ ≡ 0). (a): Shown in black is the coupling function Eq (12) with $\varepsilon_{U}=0.8$ and $\delta_{U}=\pi /8$. Shown in grey is a generic coupling function Eq (11) with $\varepsilon_{U}=0.1$, $\varepsilon_{C}=0.2$, *ε*_*F*_ = 0.7, $\delta_{U}=\pi /8$, $\delta_{C}=6\pi /5$ and *δ*_*F*_ = 1.7*π*. Vertical lines indicate the phase points $\theta =\delta_{U}+\pi$ (red) and *θ* = *δ*_*F*_ (blue). Solid dots indicate the stable fixed points and open dots indicate the unstable fixed points of the respective dynamics. (b): Shown is an angular coupling function *G*_*h*_(.) where $g\left(\theta -\delta_{C}\right)=\varepsilon_{C}\text{sin}\left(2\left(\theta -\delta_{C}\right)\right)$. The parameters are $\varepsilon_{U}=0.3$, $\varepsilon_{C}=0.5$, *ε*_*F*_ = 0.1, $\delta_{U}=\pi /8$, $\delta_{C}=6\pi /5$ and *δ*_*F*_ = 0.7*π*. Due to the second harmonics, the dynamics allows for two stable orientations.

We can check this for example for the simplified angular dynamics

$$\begin{array}{c} {\dot{\theta }}_{j}=g\left(\theta -\delta_{U}\right)=\varepsilon_{U}\text{sin}\left(\theta_{j}-\delta_{U}\right)\;, \end{array}$$ (12)

in which we assume that tumbling is absent (*L*_*j*_ ≡ 0) and that jellyfish orient just due to the flow of the water. In this case, there exist exactly two fixed points $\theta_{j}^{⋆}=\delta_{U}$ and $\theta_{j}^{⋆}=\delta_{U}+\pi$ at which the orientation remains unchanged (${\dot{\theta }}_{j}=0$, see panel (a) Fig 2). The first point corresponds to swimming in the direction of flow and is by construction unstable. This means that small external perturbation of orientation will grow in this point such that the jellyfish rotate out of alignment. The second point however, is stable and corresponds to swimming against the local direction of flow. For the remaining two orientational inputs, a similar rational applies yielding $\theta_{j}^{⋆}-\delta_{C}=\pi$ (vorticity) or $\theta_{j}^{⋆}-\delta_{F}=0$ (prey), corresponding to avoidance of turbulence and a directed swimming towards prey respectively.

The coupling function Eq (11) gives rise to exactly one stable orientation $\theta_{j}^{⋆}$ and its value depends on the coupling parameters $\varepsilon_{\left(U,C,F\right)}$ and orientations $\delta_{\left(U,C,F\right)}$. For example, when the coupling function *G*(.) is dominated by coupling to prey ($\varepsilon_{\left(U,C\right)}⪡\varepsilon_{F}$), agents mainly orient towards prey such that $\theta_{j}^{⋆}\approx \delta_{F}$ (grey line in (a) Fig 2). However, it is conceivable that high-order coupling terms are present in the angular dynamics as well. These terms give rise to several permissible orientations of swimming. For example, we can replace $g\left(\theta -\delta_{C}\right)=\text{sin}\left(2\left(\theta -\delta_{C}\right)\right)$ in Eq (11) to obtain a high-order coupling function (denoted by *G*_*h*_(.), see panel (b) Fig 2). When noise is present (*L* ≠ 0), jellyfish will be perturbed out out their stable orientations. In that case, it depends on the amplitude of the coupling constants how likely it is to stay in the direction of a stable equilibrium point. If the dynamics is multi stable, agents can switch in between different equilibria [122]. We exploit this fact to introduce directional decision making by allowing for parametric switches:

$$\begin{array}{cc} \varepsilon_{U}\left(F_{j}\right) & =\varepsilon_{U,0}\left[1-R\left(1,f_{U},F_{j}\right)\right],\;\varepsilon_{\left|C\right|}\left(A_{j}\right)=\varepsilon_{|C|,0}\left[1-R\left(1,f_{\left|C\right|},A_{j}\right)\right],\\ \varepsilon_{F}\left(A_{j}\right) & =\varepsilon_{F,0}R\left(1,f_{F},A_{j}\right)\left[1-R\left(1,f_{g},A_{j}\right)\right]\;. \end{array}$$ (13)

Here, $\varepsilon_{\left(U,C,F;0\right)}$ are the respective angular coupling parameters in inactive (U,|C|) or active (*F*) state. In an inactive state, agents orient against the flow and avoid turbulent regions. An increase of activity will initiate the following cascade of events:

- The pulsation frequency $\omega_{j}\left(A_{j}\right)$ increases alongside the amplitude of bell strokes and the coefficient of angular diffusion $D(A_{j})$, causing an increase in the speed oscillations of swimming and a more frequent change of the swimming direction.
- Jellyfish lose interest in turbulence avoidance. Instead, they initially favor orientations towards high concentrations of prey.
- In response to increase in the instantaneous prey concentration, individuals gradually lose their orientation against the flow.
- When a jellyfish reaches high levels of activity, it again loses its interest in swimming towards prey and establishes a state of free floating.

Additional influence on the orientation can arise due to the presence of other agents, resulting in further structure formation according to more interaction terms $g_{j}\left(x_{k},x_{j},\theta_{k},\theta_{j},A_{j}\right)$ in Eq (11) that depend on the positions, orientations and distance of respective agents *j* and *k* [46, 59, 123, 124]. To the best of our knowledge, there are no quantitative estimates for a directional interaction function in jellyfish, hence for now we do not incorporate these effects in the model. We keep in mind however that in dense swarms, probably mostly during the reproduction periods, the mutual interaction between the agents may play a dominant role.

#### Positional dynamics

We consider an over-damped positional dynamics of the form:

$$\begin{array}{cc} {\dot{x}}_{j} & =v_{j}\left(X,\theta ,\varphi ,A_{j},F_{j},U_{j},t\right)=U_{j}+V_{j}\left(\varphi_{j},U_{j},A_{j},\theta_{j}\right)\\ & +\frac{1}{N_{j}}\sum_{k\neq j}^{N_{j}}I_{\text{attr}}\left(x_{k},x_{j},\varphi_{k},\varphi_{j},\theta_{k},\theta_{j}\right)+I_{\text{rep}}\left(x_{k},x_{j},\varphi_{k},\varphi_{j},\theta_{k},\theta_{j}\right)+F_{\text{ext}}\left(t\right)\;. \end{array}$$ (14)

The first two terms on the right hand side account for advection and active self-propelled motion, whereas the summations account for the swarm-internal velocity component due to the attraction and repulsion between jellyfish agents. The last term addresses external forcing mainly due to the effect of confinements or obstacles.

We model the active velocity of a jellyfish by:

$$\begin{array}{cc} V_{j} & =V\left(\varphi_{j},U_{j},A_{j}\right)\hat{e}\left(\theta_{j}\right)\\ V(\varphi_{j},U_{j},A_{j}) & =(V_{0}+R(V_{a},V_{b},|U_{j}\left|+A_{j}\right))\beta \left(\varphi_{j}\right)\\ \beta (\varphi_{j}) & =\text{exp}[J(\text{cos}\left(\varphi_{j}\right)-1)]\;. \end{array}$$ (15)

**Fig 3:**
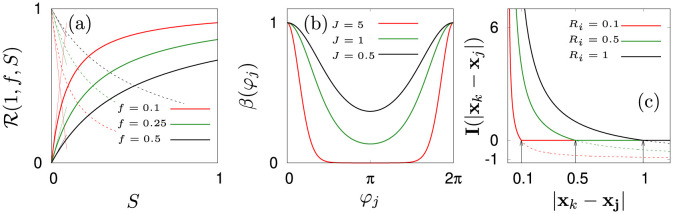
(a): Exemplary response functions Eq (5). Bold lines correspond to parameter responses of bell frequency $\omega_{j}\left(A_{j}\right)$, angular diffusivity $D(A_{j})$ and counter-current speed. Dashed lines indicate the responses of *ε*_**U**_(*F*_*j*_) and $\varepsilon_{C}\left(A_{j}\right)$ which are given by 1-R(.). Thin straight lines correspond to the quasi-linear responses with slopes of *f*^−1^ where parameters are *f* ∈ [0.1, 0.25, 0.5]. (b): The bell pulsation function *β*(*φ*_*j*_) for parameter *J* ∈ [5, 1, 0.5]. (c): Radial component of the pairwise interaction force Eq (16). Bold lines show the soft-core repulsion used in this study. Dashed lines indicate the omitted attractive part of the interaction. Black arrows indicate the respective cutoff radii at *R*_*i*_ = [0.1, 0.5, 1] m.

Here, $\hat{e}=\left[\text{cos}\left(\theta_{j}\right),\text{sin}\left(\theta_{j}\right)\right]$ is the orientation vector of agent *j*, *V*_0_ is the maximal propulsion speed of the jellyfish in their inactive state and parameters *V*_*a*_, *V*_*b*_ characterize speed adaptions due to changes of current and activity. We parameterize the bell oscillation explicitly. Our choice is inspired by the results found in [4, 26, 30, 125–127] and many other publications on jellyfish swimming which all report or assume pulsed swimming based on varying types of bell movement. In this paper, we use a simplified symmetric version of bell movement that can be adjusted by shape parameter *J* (see Fig 3(b)). When *J* is small the self propulsion never fully vanishes, as is the case for species that benefit from inertial effects. In contrast, when *J* is large, jellyfish temporarily come to a halt as can be observed in species that have developed certain types of jet propulsion.

Attraction and repulsion of agents are taken into account by the terms **I**_attr_(.) and **I**_rep_(.), where the dependencies on *θ* and *φ* allow to incorporate physiological abilities of single medusae to sense and communicate in a swarm and in a complex environment. Nonetheless, due to the lack of experimental data on these dependencies we keep both **I**_attr_(.) and **I**_rep_(.) to be only functions of relative distances. Moreover, since observational evidence for attraction of agents is limited, we assume that the active agents are soft spheres and restrict the interaction to within a radius of *R*_*i*_ (see Fig 3(c)) [47, 57, 77, 123, 128, 129]. Accordingly, *N*_*j*_ is the number of agents found within an interaction distance of *R*_*i*_. The resulting repulsion corresponds to unidirectional sensing and communication:

$$\begin{array}{c} I_{\text{attr}}\left(x_{k},x_{j}\right)=\frac{x_{k}-x_{j}}{|x_{k}-x_{j}|},\;I_{\text{rep}}\left(x_{k},x_{j}\right)=-R_{i}\frac{x_{k}-x_{j}}{|x_{k}-x_{j}{|}^{2}}\;. \end{array}$$ (16)

We denote the resulting summation of contributions in Eq (14) by **I**(**x**_*j*_).

The type of interaction represents a fluid-like repulsion while studies on active colloids often consider molecular interactions (for instance Lenard-Jones potentials) [77, 129–131]. We choose this approach because jellyfish are soft and most of the time avoid bumping into each other such that the interaction involves only the fluid. As such, the given interaction resembles the leading order terms of a flow field around an active swimmer on the micro scale [64, 132]. Thus, the repulsion **I**(**x**_*j*_) only partly represents the turbulent fluid dynamics of jellyfish swimming and we rather see the coupling as a computationally less costly parameterization of the true physical interaction, similar to [43].

Finally, the only external force **F**_ext_ accounts for interactions with obstacles. In our case, we mimic the avoidance of walls [75, 133] in a tank. We model the wall forces by a one-dimensional soft-sphere repulsion, similar to Eq (16) with a repulsion radius of 0.1 m. A simplistic comparison of the overall model and already existing research on active matter and jellyfish prediction models is shown in Table 1.

## Discussion and results

**Fig 4:**
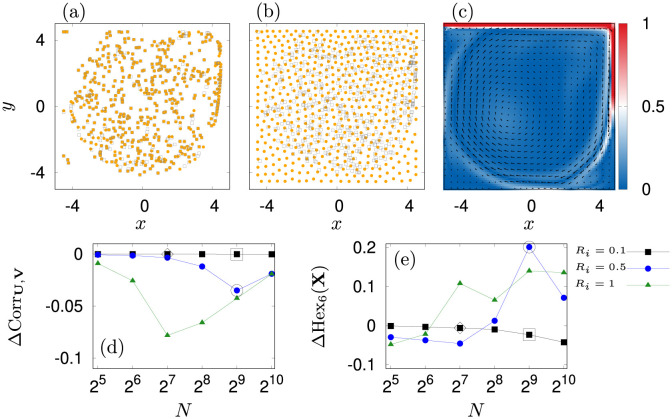
Results for a cavity flow at Re = 10^5^ and for *N* = 512 agents. The upper wall moves at a constant speed of 0.4 m s^−1^ to the right. (a): Swarm of repelling jellyfish (dots) for *R*_*i*_ = 0.1 m. Passive particles are indicated by squares and almost overlap with the position of their active counterparts (orange). (b): A similar swarm but now for *R*_*i*_ = 0.5 m. In this setting the swarm is heavily influenced by the confinement. (c): Flow field **U**(**x**, *t*) (arrows) and absolute vorticity |C|(x,t) (values higher than unity are cut off) on a domain of 10 m × 10 m. (d): Difference in velocity correlation of active and passive swarm for *R*_*i*_ ∈ [0.1, 0.5, 1] m (squares, dots, triangles). (e): The corresponding difference of hexatic order parameters for averaging over the 6 nearest neighbors. Marked squares and circles correspond to panel (a) and (b) respectively. Diamonds mark the parameter setting chosen in this paper.

**Fig 5:**
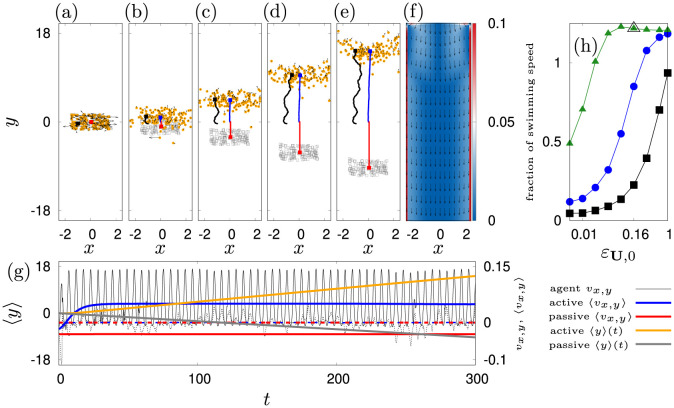
Simulation results of counter-current swimming in a horizontal channel flow at Re = 4500. The peak inflow velocity is set to 0.045 m s^−1^. Positions of active (orange) and passive (grey) agents at (a): *t* = 0 s, (b): *t* = 30 s, (c) *t* = 100 s, (d): *t* = 200 s and (e): *t* = 300 s for *ε*_**U**,0_ = 0.16 rad s^−1^, λ_*θ*_ = 5 s^−1^, *D*_0_ = 0.1 rad^2^ s^−3^, *J* = 1, *V*_0_ = 0.15 m s^−1^, *V*_*a*_ = 0.5 m s^−1^ and *V*_*b*_ = 0.6 m s^−1^. Velocities of active agents are indicated by arrows. An exemplary agent trajectory is shown in black. Blue and red trajectories indicate the center of mass movement of active and passive agents. (f): Flow field **U**(**x**, *t*) (arrows) and absolute vorticity |C|(x,t) (color, values are cut off at 0.1 rad s^−1^) on a lattice of 41 × 321 points (5 m × 40 m) (g): Averaged trajectories 〈*y*〉(*t*) (left ordinate) for passive (grey) and active (orange) agents. Corresponding velocities are shown on the right ordinate. Dashed lines indicate the *x*-components of velocity, bold lines show the *y*-components. The thin black lines indicate the velocities of the exemplary agent in panels (a-e). (h): Fraction of swimming speed as a function of the orientation strength for *D*_0_ = 0.1 rad^2^ s^−3^ and λ_*θ*_ ∈ [0.2, 1, 5] s^−1^ in black, blue and green respectively. The optimal parameter set is indicated by a bigger triangle.

We consider three paradigmatic flow environments in a rectangular tank that allow us to compare the performance of our jellyfish model with observational findings. In the first setting, we simulate a cavity flow driven by the movement of the upper wall boundary from the left to the right (see panel (c) of Fig 4). We fix the wall velocity to 0.4 m s^−1^ [134]. The second setting is a channel flow in a tank of 5 m in width and 40 m in length (see panel (f) of Fig 5). The fluid enters the domain at +20 m and leaves the domain at −20 m. We assume a fully developed Poiseuille flow at the inlet with a magnitude of −0.045 m s^−1^ in accordance with experimental parameters in [23]. In the third setting we simulate a cavity flow in a tank of 10 m width and 5 m length. We let the left and right wall of the tank move upwards at a speed of 0.4 m s^−1^. This way we generate two counter rotating main gyres that separate the domain dynamically into two parts. Throughout all of our simulations we use fluid parameters given in Table 2. Thus, the Reynolds numbers of the cavity flows are Re = 10^5^ and for the channel flow it is Re = 4500.

**Table 2:** Parameters of the jellyfish swarming model Eqs (3)–(11) and Eqs (13)–(16) and fluid parameters.

Phenomenon	Parameters [unit]	Value	External inputs
Attraction and repulsion	*R*_*i*_ [m]	0.1	–
Phase diffusion	λ_*θ*_ [s^−1^]	**5**	–
	*D*_0_ [rad^2^ s^−3^]	0.1	
Swimming against the flow	*V*_0_ [m s^−1^]	**0.15**	Flow field **U**_*j*_
	*V*_*a*_ [m s^−1^]	**0.5**	
	*V*_*b*_ [m s^−1^]	**0.6**	
	*J* [–]	1.0	
	*ε*_**U**,0_ [rad s^−1^]	**0.16**	
Avoidance of turbulence	$\varepsilon_{C,0}$ [rad s^−1^]	**0.08**	Gradient of absolute Vorticity ${\nabla |C|}_{j}$
Bell oscillations	*ω*_*j*,0_ [rad s^−1^]	1.2	–
Prey search behavior	$\lambda_{A}$ [s^−1^]	0.005	Prey concentration *F*_*j*_
	*ε*_*F*,0_ [rad s^−1^]	**0.16**	Prey gradient ∇*F*_*j*_
	*f*_*φ*_ [–]	0.75	
	*f*_*θ*_ [–],	0.75	
	*f*_**U**_ [*F*_0_]	**0.05**	
	$f_{C}$ [–]	**0.2**	
	*f*_*F*_ [–]	**0.2**	
	*f*_*g*_ [–]	100	
Fluid dynamics parameters			
Density 1000 kg m^3^	Effective viscosity 0.4 Ns m^−2^		
Prey diffusivity *D*_*F*_ = 0.001 m^2^ s^−1^			

Parameters estimated in this study are shown in bold letters. The fluid time step is 0.001 s. Agent and fluid variables are stored every 0.1 s. *F*_0_ is the saturation level of prey in the fluid.

### Wall effects

Here, we first discuss what effects arise due to repulsive interactions at walls ($F_{\text{ext}}$) and due to agent-agent interaction ($I(x_{j})$ in Eq (14). For this, we simulate two ensembles with reduced dynamics

$$\begin{array}{c} {\dot{x}}_{j}=U_{j}+F_{\text{ext}},\;\text{and}\;{\dot{x}}_{j}=U_{j}+I\left(x_{j}\right)+F_{\text{ext}}\;. \end{array}$$ (17)

The first ensemble mimics passive tracers. They get advected by their local fluid velocities **U**_*j*_ and they are confined by the wall forces **F**_ext_. In the second ensemble, also volume exclusion is present due to **I**(**x**_*j*_). It is well known that in the presence of volume-excluding agent-agent interactions, states of (quasi-) periodic spacial order may emerge [57, 135, 136]. We seek to avoid such states as they would mask the structure formation process due to swimming and orientation in our simulations. In our model, the transition towards ordered states is influenced by the interaction radius *R*_*i*_ and by the number of agents in the domain, which we have to select accordingly.

First, we set the interaction distance at walls for this experiment to 0.5 m. Example results are depicted in Fig 4. We calculate the correlations of velocity and the hexagonal order parameter

$$\begin{array}{c} {\text{Corr}}_{V,U}=\frac{1}{2}[{\text{Corr}}_{\dot{x},U_{x}}+{\text{Corr}}_{\dot{y},U_{y}}],\;{\text{Hex}}_{n}\left(X\right)={\bar{\;|{\bar{\text{exp}(i6\delta )}}_{n}|\;}}_{N} \end{array}$$ (18)

for the active and passive tracers. To obtain the local hexagonal order, we find for each agent *j* its *n* nearest neighbors. Then, *δ* is the relative angle between agents *j* and *k* (obeying tan(*δ*_*j*.*k*_) = (*y*_*j*_ − *y*_*k*_)/(*x*_*j*_ − *x*_*k*_)) and the complex order parameter is given by an average of exp(*i*6*δ*_*j*,*k*_) over these *n* nearest neighbors. To obtain the ensemble order, we finally average over the ensemble of *N* agents [136, 137]. The velocity correlation measures how much the ensemble follows the background flow while the hexagonal order parameter quantifies local lateral order. For example if the agents were to assemble in perfect hexagons, angles of the 6 nearest neighbors *δ*_*j*,*k*_ = *π*/6 inside the domain. Accordingly, Hex_6_(**X**) = 1. If any other symmetry would arise or any other disordered state, it would be indicated by a certain lower level of Hex_6_(**X**).

We see that the swarm undergoes a phase transition from almost free floating to a disordered crystal phase (panels (a) and (b) in Fig 4), depending on the parameters *R*_*i*_ and *N*. This can be observed in Fig 4(d) and 4(e) for the time averages of ΔCorr_**U**,**V**_ = 〈〈Corr_**U**,**V**_ − Corr_**U**,**V**;passive_〉〉_*t*_ and ΔHex_6_(**X**) = 〈〈Hex_6_(**X**) − Hex_passive,6_(**X**)〉〉_*t*_ after an initial transient of 50 s.

At small swarm sizes the agents essentially follow inertial trajectories. When the number of agents increases, we see that for larger interaction radii the velocity correlation drops due to swarm-internal repulsive movements. Depending on *R*_*i*_, the particles essentially lose their ability to float freely. However, at sufficiently large area densities, agents are so closely packed that local repulsive interactions cancel each other (see S1 Movie). Since the positional dynamics Eq (17) is over-damped, it means that particles are still tied to their local fluid velocities such that their motion gains back its correlation with the local flow [135].

In parallel, hexagonal order reveals that for *R*_*i*_ = 0.1 m, passive and repulsive swarm behave essentially similarly. At high area densities, we observe that repulsive movement causes a decrease in relative order. On the contrary, we see that the order shows a maximum before it decreases again for larger repulsion radii. This is because the natural increase in hexatic symmetry is disrupted by the flow perturbations and by the quadratic symmetry of the boundary which infiltrates into the domain once long range correlations establish due to dense packing.

Based on this discussion, we set the number of agents to *N* = 128 and the repulsion radius to *R*_*i*_ = 0.1 m as these parameters guarantee almost free floating of the jellyfish-like particles.

### Swimming against the flow

We are in particular interested in the swimming dynamics of the jellyfish *Rhopilema nomadica*. For this species, the ability to counteract the underlying current has been measured in a long channel at different inflow velocities [23]. The swimming speed in the absence of a flow was estimated to 0.067 m s^−1^ and an active swimming speed of 0.083 m s^−1^ was reported for an inlet velocity of 0.045 m s^−1^.

We adapt the model of passive-repulsive tracers Eq (17) by allowing for an angular dynamics affected by angular diffusion and orientation against the flow in Eq (11), a non-zero swimming speed according to Eq (15) and bell pulsation Eq (6), resulting in the model:

$$\begin{array}{c} {\dot{x}}_{j}=U_{j}+V\left(\varphi_{j},U_{j}\right)\hat{e}\left(\theta_{j}\right)+I\left(x_{j}\right)+F_{\text{ext}},\\ {\dot{\varphi }}_{j}=\omega_{j,0},\;{\dot{\theta }}_{j}=L_{j}+\varepsilon_{U,0}\text{sin}\left(\theta_{j}-\delta_{u}\right)\;. \end{array}$$ (19)

Panels (a-e) Fig 5 show snapshots of the resulting swarm dynamics. S2 Movie depicts the time evolution of the swarm.

The, swimming dynamics is defined by the parameters λ_*θ*_ (inverse of the correlation time for the angular noise), *D*_0_ (amplitude of the angular white noise), *V*_0_ (amplitude of swimming speed in the inactive state), *V*_*a*_, *V*_*b*_ (velocity response parameters), *ε*_**U**,0_ (angular coupling constant) and *J* (shape parameter of velocity oscillations). Here we adjust parameters of the model to experimental results [23]: We choose *J* = 1 to mimic inertia of medusae during a bell stroke (see Fig 3(a)). To obtain other parameters, we assume that agents are fully aligned against the flow and we average the orientation dynamics over time. Then, the oscillatory component *β*(*φ*) results in a factor of roughly 0.47 and other parameters follow directly: *V*_0_ = 0.15 m s^−1^, *V*_*a*_ = 0.5 m s^−1^ and *V*_*b*_ = 0.6 m s^−1^.

The corresponding averaged swimming velocity is 0.087 m s^−1^. We compensate the excess by allowing for angular diffusion in the simulations. Then, the dynamics depends on parameters *ε*_**U**,0_, λ_*θ*_ and *D*_0_. We fix *D*_0_ = 0.1 rad^2^ s^−3^ and let λ_*θ*_ ∈ [0.2, 1, 5] s^−1^ ($\sigma^{L}=\sqrt{D_{0}/\lambda_{\theta }}\in \left[0.71,0.32,0.14\right]$ rad s^−1^). We perform a parameter scan for *ε*_**U**,0_ ∈ [0.005⋅2^(0, 1, …7)^, 1] rad s^−1^ and compute the time average of $\left|\langle \dot{x}\rangle -U\right|$ in the central two third of the simulation time (see panel (g) Fig 5). We divide the average by 0.067 m s^−1^ to obtain the fraction of swimming speed in presence of a current, similar to the analysis in [23]. Panel (h) in Fig 5 depicts the transition from noisy advection to active counter-current swimming. Our agents swim 21% faster against the flow at *ε*_**U**,0_ = 0.16 rad s^−1^ and for λ_*θ*_ = 5 s^−1^ (Fig 5 bigger triangle in panel h).

The essence of the counter-current swimming can be understood by means of the angular dynamics in Eq (19). We see that the noise has a standard deviation of *σ*_*L*_ such that we expect the existence of the fixed point solution $\theta_{j}^{⋆}-\delta_{u}=\pi$ to become more likely when *ε*_**U**,0_ > *σ*^*L*^. For the given parameter, this critical average coupling is *σ*^*L*^ = 0.14 rad s^−1^ which indeed is slightly beyond the transition zone of panel (h) Fig 5. In turn, phase slips across the fixed point solution when the noise is temporarily too strong, can still occur. Then, the agents start to tumble or even rotate for some time such that their effective propulsion speed against the flow is drastically reduced (see S3 and S4 Movies). From panel (h) Fig 5 it can be seen that such events are unlikely before the fixed point threshold is reached. Thus, we consider the reconstructed set of parameters to be optimal as it stipulates the frequently encountered phenomenon of *criticality* in neuronal responses and biological systems in general [138, 139].

### Turbulence avoidance and structure

The avoidance of turbulent regions in a flow can be implemented in a straight forward way by a second alignment term in Eq (19) of strength $\varepsilon_{C,0}$:

$$\begin{array}{c} {\dot{\theta }}_{j}=L_{j}+\varepsilon_{u,0}\text{sin}\left(\theta_{j}-\delta_{u}\right)+\varepsilon_{C,0}\text{sin}\left(\theta_{j}-\delta_{C}\right)\;. \end{array}$$ (20)

**Fig 6:**
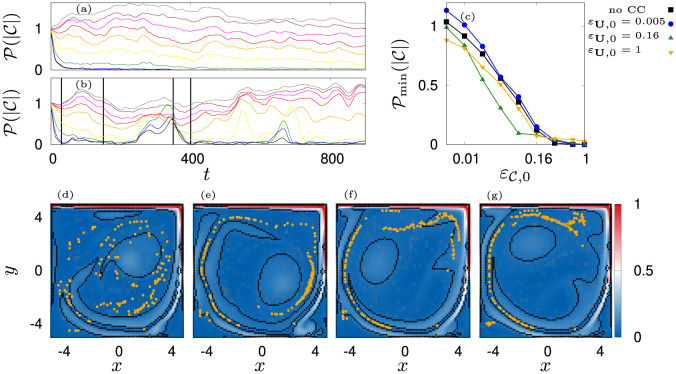
Depicted are the time courses of turbulence fraction P(|C|) given (a): *ε*_**U**,0_ = 0.005 rad s^−1^ and (b): *ε*_**U**,0_ = 1 rad s^−1^ for $\varepsilon_{C,0}\in \left[0.005\cdot 2^{\left(0,\ldots ,7\right)},1\right]$ rad s^−1^ in grey, brown, magenta, red, orange, yellow, green, blue and black respectively. Panel (c) depicts the averaged baseline turbulence fraction for four modes of counter-current swimming. The flow domain and the position of active agents for $\varepsilon_{C,0}=0.32$ is shown in (d): *t* = 30 s, (e): *t* = 150 s, (f): *t* = 350 s, (g): *t* = 400 s, also indicated by black vertical lines in panel (b). Characteristic regions are indicated by black boundaries. The vorticity is indicated with a color scale.

This second term causes the agents to also orient against the gradient of absolute vorticity and thus to swim away from regions of turbulence. As a consequence, flow structures are imprinted on the swarm orientation and can cause the formation of filaments and patches (see panels (d-g) in Fig 6 and S5 Movie).

We quantify the success in avoiding turbulent regions by means of the indication number Eq (1) and define the fraction:

$$\begin{array}{c} P\left(|C|\right)=\frac{N(|C|)}{N(|C{|}_{\text{passive}})}\;. \end{array}$$ (21)

When the agents all avoid turbulence, P(|C|)=0, when agents are initially randomly distributed and the active agents all reside inside areas of high turbulence, $P=1/N(|C{|}_{\text{passive}})$. The characteristic regions where $|C|>{\bar{\left|C\right|}}_{\text{passive},N}$ are indicated by the black boundary in Fig 6. We see that these regions for the cavity flow evolve essentially into two substructures, an elliptic central region and a long and narrow hose-like structure generated by the clockwise main current.

Mainly influenced by these flow structures, there appear essentially two classes of transients. First, when counter-current swimming is switched off or if the orientation against the flow is weak (*ε*_**U**_ = 0.005 rad s^−1^), the number of agents in turbulent regions decreases with increased $\varepsilon_{C}$ and the transition out of turbulence takes place essentially in a short initial time interval (see Fig 6 panels (a)). Second, when counter-current swimming is sufficiently strong, the jellyfish effectively stand still in the water or achieve a net velocity against the flow. In that situation, they stay in a surrounding turbulence structure for a prolonged period of time and can group along this structure into patches or long filaments (see Fig 6 panels (d-g), S5 Movie). Nevertheless, the swarm can eventually encounter turbulent regions in which it stays for some time before it starts avoiding these regions (see Fig 6 panel (f)). When this happens, we observe intermittent spikes in P(|C|) that decrease in amplitude before the swarm has adjusted to the turbulence structures (see Fig 6 panel (b)).

Since spikes are isolated and transient events in time, we first omit the initial 50 s and calculate the maximal and minimal fractions $\hat{P}\left(|C|\right)$ and $\tilde{P}\left(|C|\right)$. Then, we take into account only values $P\left(|C|\right)<0.9\tilde{P}\left(|C|\right)+0.1\hat{P}\left(|C|\right)$ to obtain the time average $P_{\text{min}}\left(|C|\right)$ (see Fig 6 panel (c)). The resulting curves show that the baseline avoidance of turbulence is essentially independent of *ε*_**U**,0_.

Additionally, we characterize the pattern formation by ΔHex_12_(**X**) = 〈〈Hex_12_(**X**) − Hex_passive,12_(**X**)〉〉_*t*_ and the difference of standard deviation ΔΣ = 〈〈Σ(**X**) − Σ_passive_(**X**)〉〉_*t*_ where

$$\begin{array}{c} \Sigma \left(X\right)=\sqrt{\frac{{\left(\sigma^{x}\right)}^{2}}{2{\left(\sigma^{x}\right)}^{2}\left(0\right)}+\frac{{\left(\sigma^{y}\right)}^{2}}{2{\left(\sigma^{y}\right)}^{2}\left(0\right)}}\;. \end{array}$$ (22)

Here, *σ*^*x*^ and *σ*^*y*^ are the standard deviations of the agents positions in *x* and *y*-direction respectively at time *t*. At *t* = 0, Σ(**X**) = 1 and stays approximately constant for the passive swarm while it increases or decreases according to the evolution of the active swarm. We simulate the dynamics for 900 s and take into account values in the last 300 s for time averaging. In contrast to the analysis of regular structural order, we have chosen 12 nearest neighbors for averaging of the phase factor exp(*i*6*δ*) in Eq (18), because it allows us to better resolve the effect of filamentation.

**Fig 7:**
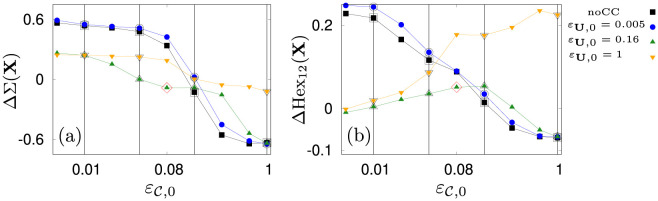
Measures of structure formation. (a): Spread of the swarm ΔΣ(**X**), (b): Local hexagonal order ΔHex_12_(**X**) for four settings of counter-current swimming. Shown as vertical lines are columns in Fig 8 for $\varepsilon_{C,0}\in \left[0.01,0.04,0.16,1\right]$ rad s^−1^. The red diamond indicates the optimal coupling constant for turbulence avoidance ($\varepsilon_{U}=0.16$ rad s^−1^, $\varepsilon_{C}=0.08$ rad s^−1^).

**Fig 8:**
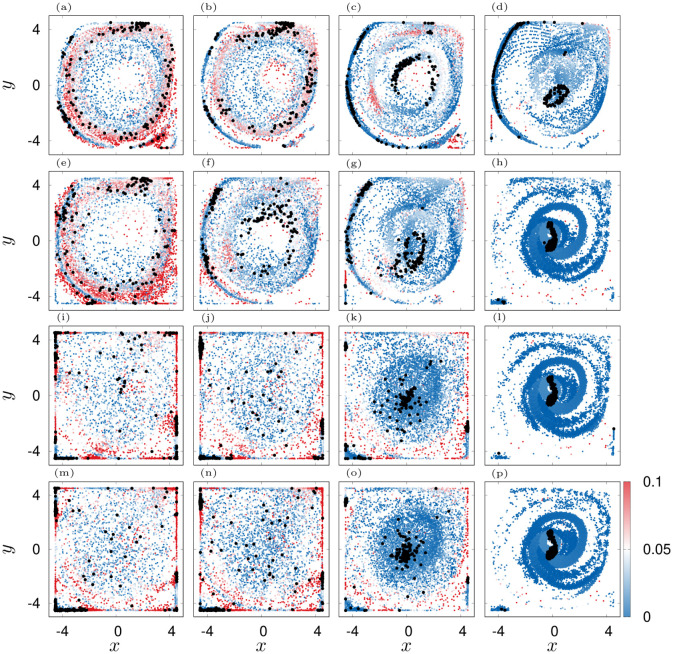
Scatter plots of agents position over 900 s. Color indicates |C|. The final configurations are shown with black dots. Panels (a-d): *ε*_**U**,0_ = 1 rad s^−1^, (e-h): *ε*_**U**,0_ = 0.16 rad s^−1^, (i-l): *ε*_**U**,0_ = 0.005 rad s^−1^, (m-p): no counter-current swimming. Columns from left to right correspond to $\varepsilon_{C,0}=\left[0.01,0.04,0.16,1\right]$ rad s^−1^ respectively.

We observe that the structure formation process heavily depends on the combination of $\varepsilon_{C,0}$ and *ε*_**U**,0_. When swimming against the flow is switched off or if it is weak, the jellyfish follow scattered trajectories and mostly get trapped inside the corners of the domain, causing a large spread and a relatively high order of the swarm due to compression at the walls (Fig 7 black and blue curves and Fig 8 panels (i, j, m, n), S6–S9 Movies).

**Fig 9:**
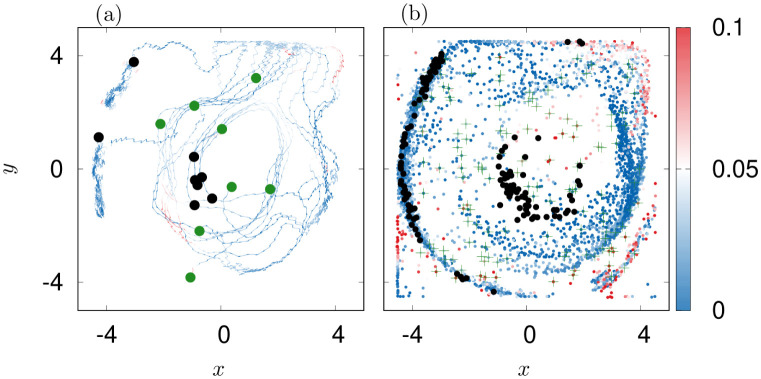
Optimal swarming dynamics for $\varepsilon_{U}=0.16$ rad s^−1^, $\varepsilon_{C}=0.08$ rad s^−1^. (a): starting positions (green) and final positions (black) of 8 exemplary jellyfish. The trajectories of the particles are shown in color. The color code indicates the encountered amplitude of absolute vorticity. (b): Scatter plot of all particles similar to Fig 8. Additionally shown are green crosses for the position of the agents at *t* = 0 s.

With increased $\varepsilon_{C,0}$ the swarm transitions into a single patch in the center (Fig 8 panels (h, k, l, o, p), S10–S14 Movies) which has a minimal spread and a minimal order. The latter is a consequence of the constant regrouping due to agents that bump into the cluster and advection. On the contrary, when the agents orient more against the flow, they are able to group into long lasting and coexisting coherent structures, namely filaments (Fig 8a–8g, S15–S21 Movies) and rings (Fig 8a, 8b, 8e and 8f, S15–S20 Movies). Most prominent is the transition for the optimal, *ε*_**U**,0_ = 0.16 rad s^−1^. For this setting, the swarm undergoes a transition from ring patterns over filaments towards patches. This transition is indeed signified by two jumps in the spread ΔΣ and an increase of local order before the system settles into the disordered patch (see green lines in Fig 7). In contrast, for *ε*_**U**,0_ = 1 rad s^−1^, the swarm transitions later from ring patterns into filaments. Based on this investigation, we chose $\varepsilon_{C}=0.08$ rad s^−1^ ($\varepsilon_{U}=0.16$ rad s^−1^) as an optimal pair of coupling values (red diamond in Fig 7, see also Fig 9). It gives rise to a dynamics that sits “in between” scattered states and patches and thus, ensures a relatively rich behavior of the simulation model as already small variations of $\varepsilon_{C}$ will lead to a significant change in the swarming patterns. We again favor such a behavior as it mimics the frequently encountered phenomenon of *criticality* in biological systems [138, 139].

### Foraging

**Fig 10:**
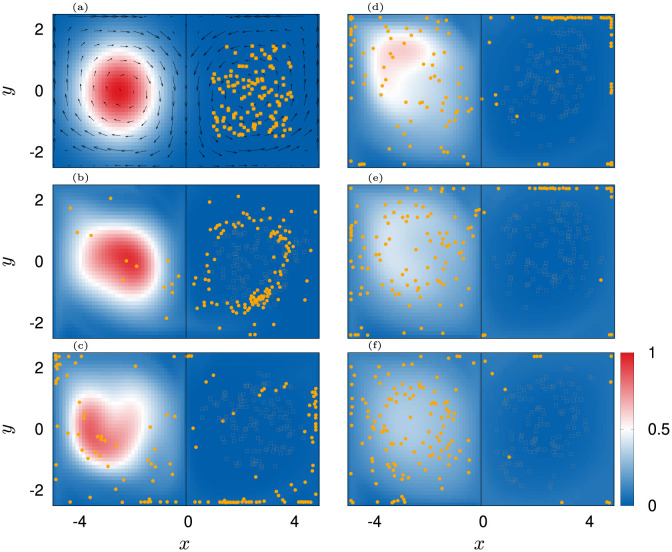
Depicted are snapshots of an optimal search behavior. The model is simulated with parameters found in Table 2. Arrows in panel (a) show the flow field. Orange dots indicate the actively swimming jellyfish. Squares indicate the passive tracers. The color map indicates the concentration of prey in the water in units of *F*_0_. (a): *t* = 0 s, (b): *t* = 30 s, (c): *t* = 100 s, (d): *t* = 300 s, (e): *t* = 600 s, (f): *t* = 900 s.

Our final goal is to capture the principal behavior of foraging based on the full dynamics Eqs (3)–(11) and (13)–(16). We simulate the swarm in a double-gyre flow in which we initialize the agents in the right gyre at *x* > 0 and the prey in the left gyre at *x* < 0 (see Fig 10 panel (a)). Thus, in this setting the jellyfish are dynamically separated from prey by a strong central current at *x* = 0 [35]. We start our model with the previously obtained optimal parameter setting of Table 2 for counter-current swimming and turbulence avoidance. Overall, each model instance runs for 900 s.

#### Response mechanisms

**Fig 11:**
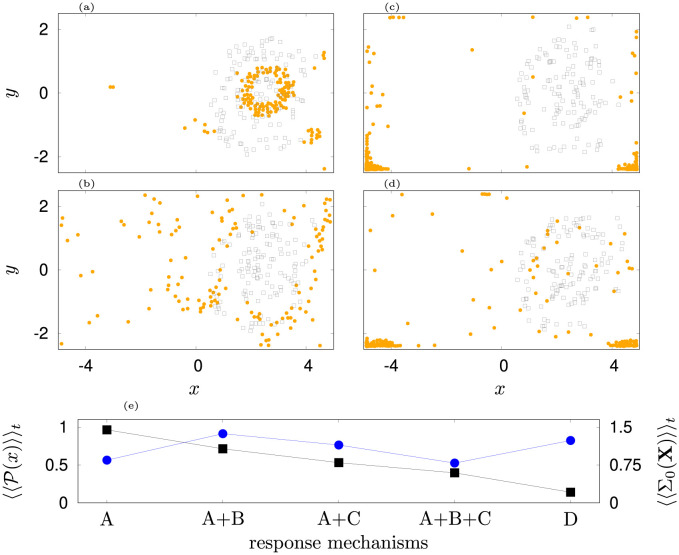
Depicted are the positions of jellyfish (orange dots) and passive tracers (grey squares) due to response mechanisms (a): A, (b): A+B, (c): A+C, (d): A+B+C. Snapshots depict the swarm in the end of the covered simulation time of 900 s. In comparison, the optimal full response is shown for scenario D in Fig 10. Panel (e) depicts the time average of agent fraction ${\langle \langle P\left(x\right)\rangle \rangle }_{t}$ that remains in the right of the tank (black squares) and the time average of the spreading of agents in the left part of the tank 〈〈Σ_0_(**X**)〉〉_*t*_ (blue dots).

The turbulence avoidance causes the swarm to form a ring structure in the right gyre of the flow (see Fig 10 panel (b) and Fig 11 panel (a)). We check first the impact of subsequent combinations of response mechanisms on the ability to search for prey:

A Higher pulsation frequency ω(A) and a larger angular diffusion D(A).
B Reduction of turbulence avoidance by means of a response $\varepsilon_{C}\left(A\right)$. We set the slope parameter for $\varepsilon_{C}$ to $f_{C}=0.02$, allowing for an almost immediate reduction when activity increases.
C Bi-linear dependence of propulsion velocity $V(U_{j}+A_{j})$.
D The full response dynamics at optimal parameters of Table 2. (This search dynamics is depicted in Fig 10 and can be observed in S22 Movie).

Prey search by jellyfish is variable but there exists experimental evidence [35, 105] and observational experience [65] that can be condensed to the following two performance criteria for our tank simulation: When jellyfish is searching successfully for prey,

- The fraction of jellyfish in the right (oligotropic) gyre is minimal, meaning that the majority of individuals manages to cross into the left domain at *x* < 0 or that individuals group in regions where prey is found.
- The jellyfish spread evenly in the left gyre once they have crossed the barrier. This mimics the free floating state when individuals shuffle water to the oral arms.

We quantify the crossing performance by means of the fraction

$$\begin{array}{c} P\left(x\right)=\frac{N_{0}\left(x\right)}{N_{0}\left(x_{\text{passive}}\right)}\;, \end{array}$$ (23)

where we indicate by index 0 that only jellyfish in the right gyre (*x* > 0) contribute ($\hat{x}=0$). Accordingly, a fraction P(x)=1 indicates that all jellyfish and all passive agents are found in the right gyre and it is zero, when all jellyfish have crossed the flow barrier. To quantify the spread of the swarm after the crossing of the barrier, we calculate Σ_0_(**X**) according to Eq (22). The index 0 indicates that for this averaging only jellyfish at *x* < 0 are taken into account. In this analysis we do not take into account the length of the transition time needed for the swarm to switch basins. However, we found that the transiton, if it takes place, is completed in the end of the simulation time.

While frequency and diffusion responses (A) do not result in significant searching (Fig 11 panel (a), S23 Movie), already enabling weakening of the turbulence avoidance (B) allows some agents to cross the flow barrier and to spread partly in the left gyre (Fig 11 panel (b), S24 Movie). A velocity response to activity increase (C) leads to a further increase in the number of crossings. However, jellyfish get trapped in the corners of the tank because direction is no longer chosen based upon turbulence avoidance and higher velocities increase the chance of bumping into a wall where the movement is constrained.(Fig 11 panel (c,d,e)), S25 and S26 Movies).

#### Interplay of directed search and counter-current swimming

Next, we also allow for an orientation into the direction of the prey. We find that this additional orientation (*δ*_*F*_) leads to complex decision making in our jellyfish model based on an interplay of directed prey search and counter-current swimming. We start our analysis by limiting the set of prey angular-coupling constant and response parameters $\left[\varepsilon_{F,0},f_{C},f_{F},f_{U},f_{g}\right]$ to symmetric switching from turbulence avoidance to prey searching ($f_{F}=f_{C}$). And, we fix the ignorance parameter *f*_*g*_ = 100, which means that even after prolonged periods of preying, resulting in high activity A, the jellyfish remain “greedy” for food. This leaves us with *ε*_*F*,0_, *f*_*F*_ and *f*_**U**_.

**Fig 12:**
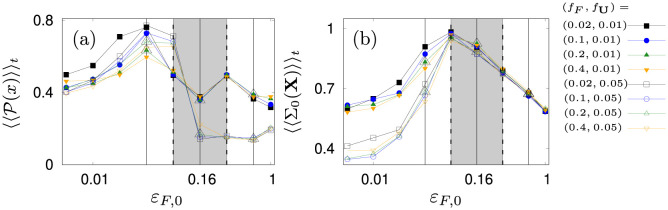
Depicted are the time averaged fraction of agents at *x* > 0, ${\langle \langle P\left(x\right)\rangle \rangle }_{t}$ in panel (a) and the spreading 〈〈Σ_0_(X)〉〉_*t*_ of agents at *x* < 0 in panel (b). Empty and filled markers correspond to *f*_**U**_ = 0.05*F*_0_ and *f*_**U**_ = 0.01*F*_0_ respectively. The grey box indicates the region of favorable *ε*_*F*,0_ rad s^−1^. Vertical black lines and larger triangles indicate parameters used in Fig 13.

**Fig 13:**
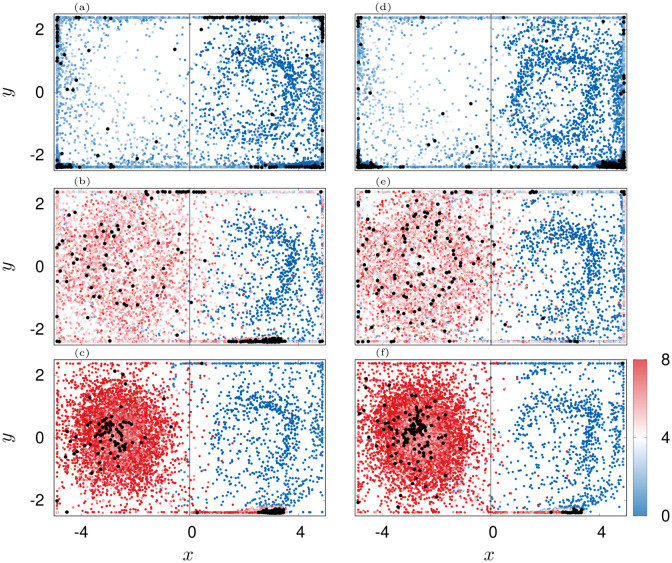
Depicted are scatter plots of jellyfish positions over 900 s and with an increment of 13 s for parameter *f*_*F*_ = 0.2. Color indicates the activity A. Black dots indicate swarm in the end of the simulation. (a): *f*_**U**_ = 0.01*F*_0_, *ε*_*F*,0_ = 0.04 rad s^−1^, (b): *f*_**U**_ = 0.01*F*_0_, *ε*_*F*,0_ = 0.16 rad s^−1^, (c): *f*_**U**_ = 0.01*F*_0_, *ε*_*F*,0_ = 0.64 rad s^−1^, (d): *f*_**U**_ = 0.05*F*_0_, *ε*_*F*,0_ = 0.04 rad s^−1^, (e): *f*_**U**_ = 0.05*F*_0_, *ε*_*F*,0_ = 0.16 rad s^−1^, (f): *f*_**U**_ = 0.05*F*_0_, *ε*_*F*,0_ = 0.64 rad s^−1^.

The ability of the swarm to cross the flow barrier is found to depend mainly on the angular coupling constant *ε*_*F*,0_ and velocity decoupling parameter *f*_**U**_. On the contrary, crossing performance is largely independent of *f*_*F*_ (see Fig 12). In particular, we see that for *f*_**U**_ = 0.05*F*_0_ (86% weakening of counter current coupling at *F*_*j*_ = 0.3 *F*_0_) more jellyfish travel to the left gyre (Fig 12 panel (a)) than for *f*_**U**_ = 0.01*F*_0_ (97% weakening of counter current coupling at *F*_*j*_ = 0.3 *F*_0_). Interestingly, this difference occurs due to the emergence of secondary clusters in the right part of the tank (see for instance panels (b,c,f) in Fig 13). These clusters emerge due to a long-time equilibrium of orientations against the flow (*δ*_**U**_) and towards prey (*δ*_*F*_). The upper cluster is less stable than the lower one because jellyfish in the upper right part of the flow are the last ones to become activated. As a consequence, they remain too slow for the flow in that region and experience a slow downstream transport. Ultimately, they come close to the upper central region of the tank where they feel the prey and cross the barrier.

On the contrary, agents of the lower cluster experience a more diffuse flow and thus, are able to counteract advection. Ultimately they are able to approach the flow barrier where an increase in activity sparks a shift in the coupling parameters such that they cross over as well. However, those jellyfish also experience a small leakage of prey from left to right domain, which is transported by the lower boundary current in which they swim. This, on the one hand causes stronger bell strokes. On the other hand, the jellyfish start to orient partly towards the ambient prey. But, since the prey gradient and the direction of the nutritious flow are mostly not aligned, many jellyfish get dragged away. Some of them find a new equilibrium position further away from the flow barrier while others get transported with the current to the upper part of the barrier where they cross. Overall, some of the jellyfish remain inside the nutritious current, mainly on the lower right side of the tank.

In fact, coupling values *ε*_*F*,0_ > 0.16 rad s^−1^ make the escape from the lower secondary cluster more difficult while at the same time causing the swarm to have a small spread in the left domain (Fig 12 panel (b)). On the contrary, for *ε*_*F*,0_ < 0.16 rad s^−1^, the jellyfish do not orient sufficiently towards prey and get trapped in the corners of the tank. This leaves us with a narrow parameter region of optimal *ε*_*F*,0_ (grey regions in Fig 12) out of which we choose *ε*_*F*,0_ = 0.16 rad s^−1^.

**Fig 14:**
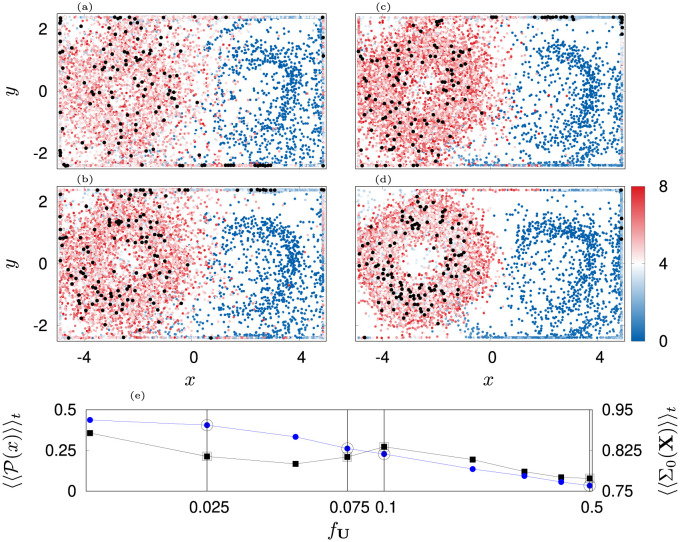
Depicted are scatter plots of jellyfish positions over 900 s and with an increment of 13 s for (a): *f*_**U**_ = 0.025 *F*_0_, (b): *f*_**U**_ = 0.075 *F*_0_, (c): *f*_**U**_ = 0.1 *F*_0_, (d): *f*_**U**_ = 0.5 *F*_0_. Other parameters are fixed according to optimal values given in Table 2. Color indicates the activity A. Black dots indicate the swarm in the end of the simulation. Panel (e) depicts the time averages of the agents fraction ${\langle \langle P\left(x\right)\rangle \rangle }_{t}$ at *x* > 0 (black squares, left ordinate) and of the spreading 〈〈Σ_0_(**X**)〉〉_*t*_ when *x* < 0 (blue dots, right ordinate).

Finally, we investigate how the response to prey depends on the velocity decoupling parameter *f*_**U**_. For this we consider just *ε*_*F*,0_ = 0.16 rad s^−1^. Here, we find that because the value of *f*_**U**_ determines the instantaneous coupling strength *ε*_**U**_(*F*), it simultaneously influences the fraction 〈P〉 of remaining agents in the right gyre and the swarm spreading in the left gyre (Fig 14 panels (a-d)). We see that for largely persistent counter-current swimming almost all agents cross the flow barrier (*f*_**U**_ = 0.5*F*_0_, 38% weakening of counter current coupling at prey concentration *F*_*j*_ = 0.3*F*_0_, Fig 14 panel (d), S27 Movie). However they do not spread in the left domain but form a ring pattern. On the contrary, for earlier loss of counter-current orientation (*f*_**U**_ = [0.025, 0.075, 0.1] *F*_0_, [92, 80, 75]% weakening of counter current coupling at *F*_*j*_ = 0.3*F*_0_, Fig 14 panel (a,b,c), S28–S30 Movies), secondary clusters remain in the right domain (Fig 14 panels (a-c)) but the ring patter in the left gyre is less pronounced. Overall, this leads us to the conclusion that we consider *f*_**U**_ = 0.05*F*_0_ as an optimal parameter as it allows almost complete crossing of the swarm and reasonable spreading of the swarm in the left domain of the tank (see Fig 10).

## Conclusion

In this paper we have proposed a paradigmatic model for jellyfish swarming based on active Brownian particles. The model can be readily incorporated in large-scale simulation models of the ocean and simulations of tank experiments. It provides a measurement paradigm to understand mechanisms of large scale structure formation based on agent responses, agent physiology and local interaction mechanisms. In its present form, Eqs (3)–(11) and (13)–(16), the model is already able to reproduce paradigmatic jellyfish behavior reported in the literature. In particular, we have been able to simulate the counter-current swimming of *R. nomadica* [23]. Using the kinematic tracer of absolute vorticity, we suggested a mechanism for the formation of long-lasting swarm patterns. Finally, we simulated the qualitatively reported behavior of jellyfish in a tank when prey is present [35, 75, 105].

Here, we will discuss several aspects of swarming that have to be investigated in a future model. We largely dismissed jellyfish physiology of swimming and agent-agent interactions. The physiology of a jellyfish comprises three aspects. First, we expect that major parts of communication among jellyfish can be attributed to the turbulent vortex rings which are send into the water with a certain angular dependence *Q*_*V*_(**x**_*k*_, **x**_*j*_, *θ*_*k*_, *θ*_*j*_) (red arrow in Fig 1 panel (a)).

Second, jellyfish possesses specific sensing capabilities. It has been reported that jellyfish occasionally bump into each other “head on” while at the same time being able to sense already faint pressure signals at a distance [65]. This suggests that jellyfish perceive their surrounding depending on the distribution of rophalia on the surface of the bell, introducing an angular dependence of sensitivity to perturbations. In the given framework, we suggest to introduce a function *Q*_*S*_(**x**_*k*_, **x**_*j*_, *θ*_*k*_, *θ*_*j*_) that corresponds to this distribution. Accordingly, the resulting physiological effect on agent-agent coupling, can be incorporated by a product function *Q*(**x**_*k*_, **x**_*j*_, *θ*_*k*_, *θ*_*j*_) = *Q*_*S*_(**x**_*k*_, **x**_*j*_, *θ*_*k*_, *θ*_*j*_)⋅ *Q*_*V*_(**x**_*k*_, **x**_*j*_, *θ*_*k*_, *θ*_*j*_). The function *Q*(.) then mimics the previously envisaged angular dependence of coupling in attraction and repulsion Eq (16) and in the functions *H*_*j*_(.) and $G_{j}\left(.\right)$, Eq (2).

Third, one needs to specify how the dynamics of the internal neuronal network reacts to external stimuli. This effect can be taken into account by the phase coupling function *H*_*j*_(.) of a medusa. It describes the response of the network phase, *φ*, Eq (2) to external perturbations and has found widespread application in research on biological oscillators [46, 70, 103, 115, 140–142]. As a consequence of the directional dependence of agent-agent coupling, given by *Q*(.), the phase response and the sensitivity response have to be disentangled simultaneously. Here, tracking techniques and phase-dynamics reconstruction have to be employed [143–145].

Currently, we have considered only a simplified fluid-like soft-core repulsion that avoids any attractive effects. Here, further investigation of the radial dependence of the flow field around a medusa are needed. Beyond the angular dependence of communication, given by *Q*(.), jellyfish might also be subject to inevitable effects of synchronization among each other if it fits their energy household [124, 146]. This effect would involve the oscillatory phase of the bell, *φ* and further modifies the attraction and repulsion in Eq (16).

Generally, synchronization effects can be subtle because biological oscillators tend to only partially synchronize. From that perspective it is an intriguing open question to which extend synchronization of bell pulsation is present in jellyfish aggregations and whether it leads to changes in swimming performance of jellyfish in blooms. For example, in our model the almost complete absence of a mechanism that could lead to synchrony of the bell oscillations causes significant performance losses of jellyfish clusters due to jamming (for instance S11 Movie). In fact, the only mechanisms through which the bell oscillations can couple in the model is present when agents search for prey. We consider this effect to be insignificant as it is caused by the interplay of prey concentration, repulsive interaction, activity dynamics and frequency response. On the contrary, bells stay effectively asynchronous such that densely packed agents push each other out of the way until they have sorted into groups where pushing becomes rare. This mechanism is know as social sorting [63]. Indeed, this effect might be partly visible in S10, S12 and S14 Movies where agents form highly dense clusters.

In our given model, walls and agents have the same effect on the positional dynamics, depending on the interaction distance *R*_*i*_. We have seen during our numerical analysis that the choice of *R*_*i*_ significantly influences the foraging of jellyfish in the secondary clusters at the walls. Thus, a further open question is the nature of the wall interactions for jellyfish.

We have completely ignored perturbations in the fluid due to the presence of agents. Our motivation here is that our model will serve as a parameterization of a sub-grid-scale process [147–149]. As such, the local fluid perturbations of single jellyfish become irrelevant. Therefore, we take the local perturbations into account only by the angular noise *L*_*j*_. One of the two essential parameters of this noise, *D*_0_, was chosen and thus, introduced a time scale to which the coupling constants of orientation relate. Moreover, we use a normalized prey concentration by introducing the maximum level of prey *F*_0_. To make the simulation more accurate, the actual statistical properties of the angular noise need to be investigated from passive observations and the prey concentration needs to be normalized according to an empiric *F*_0_. Furthermore, in a large-scale ocean simulation, the fluid-agent coupling will require a high-order interpolation scheme in time and space to provide accurate Lagrangian field values. In turn, the agent-fluid interaction shall rely on averaging over agents in a single cell.

The decision making of jellyfish in our model is realized by response functions R(.) that differ only by two parameters and are otherwise similar in shape (see panel (c) Fig 3). We have employed this concept of response functions because of its great success in the description of cell membrane responses in computational neuroscience [106, 107]. In contrast to those systems where the membrane response was measurable directly, investigation of the response parameters will have to rely on indirect observations and data fitting methods such as machine learning [72].

We have simulated a two-dimensional swarming dynamics. Thus, in its present form, the model Eqs (3)–(11) and (13)–(16) can be used to capture the vertically averaged dynamics of a swarm and the dynamics of jellyfish in periods of reduced vertical migration. However, in reality, jellyfish swimming is influenced by the day night-cycle [88, 150], its prey and environmental inputs such as temperature and salinity [13] which cause vertical migration. Augmentation of the model dynamics to three dimensions is relatively straight forward and requires a second directional angle.

We have chosen an over-damped positional dynamics Eq (14) to model the swimming. By this we have in mind, that on larger scale, in the sea, the short time and length scales associated with inertia are not relevant. However, this assumption gives rise to two effects in our work. First, we observe regaining of mobility once the density of individuals becomes large enough (see Fig 4). Second, the phase in the velocity oscillation function *β*(*φ*) Eq (15) has only minimal effect on the positional dynamics as the time-average of velocity is completely determined by the shape parameter *J*. A future model might consider also inertia for jellyfish, including their velocity dynamics [42, 53]. However, such a model requires twice as much initial conditions (for the positions **x**_*j*_(0) and velocities **v**_*j*_(0)), imposing even greater demands on any data-driven set of initial conditions.

The turbulence avoidance and the prey searching represent a type of first-passage problem in which the supremum of the first passage times of single agents could be used to define a first passage time for the swarm to enter the target region [151]. However, we would like to point out that agent trajectories are not independent of each other and that the boundary of the target region can be expected to have a fractal, time-dependent structure according to the Reynolds number.

Finally, we have investigated just a finite time horizon of maximal 15 minutes due to numerical constraints. We think that in future analysis longer observation times are needed to capture long-term effects of swarming.

## Supporting information

S1 Movie Wall effects *N* = 512, *R*_*i*_ = 0.5 m, Fig 4(b). (MP4)

S2 Movie Counter-current swimming λ_*θ*_ = 5 s^−1^, Fig 5 panels (a-e). (MP4)

S3 Movie Counter-current swimming λ_*θ*_ = 1 s^−1^, Fig 5. (MP4)

S4 Movie Counter-current swimming λ_*θ*_ = 0.2 s^−1^, Fig 5. (MP4)

S5 MovieAdaptive swimming *ε*_U,0_ = 1 rad s^−1^, $\varepsilon_{C,0}=0.32$ rad s^−1^, Fig 6 panels (d-g).(MP4)

S6 MovieTrapped swarm *ε*_U,0_ = 0.005 rad s^−1^, $\varepsilon_{C,0}=0.01$ rad s^−1^, Fig 8(i).(MP4)

S7 MovieTrapped swarm *ε*_U,0_ = 0.005 rad s^−1^, $\varepsilon_{C,0}=0.04$ rad s^−1^, Fig 8(j).(MP4)

S8 MovieTrapped swarm no counter-current swimming, $\varepsilon_{C,0}=0.01$ rad s^−1^, Fig 8(m).(MP4)

S9 MovieTrapped swarm no counter-current swimming, $\varepsilon_{C,0}=0.04$ rad s^−1^, Fig 8(n).(MP4)

S10 MoviePatch *ε*_U,0_ = 0.16 rad s^−1^, $\varepsilon_{C,0}=1$ rad s^−1^, Fig 8(h).(MP4)

S11 MoviePatch and trapped swarm *ε*_U,0_ = 0.005 rad s^−1^, $\varepsilon_{C,0}=0.16$ rad s^−1^, Fig 8(k).(MP4)

S12 MoviePatch *ε*_U,0_ = 0.005 rad s^−1^, $\varepsilon_{C,0}=1$ rad s^−1^, Fig 8(l).(MP4)

S13 MoviePatch and trapped swarm no counter-current swimming, $\varepsilon_{C,0}=0.16$ rad s^−1^, Fig 8(o).(MP4)

S14 MoviePatch no counter-current swimming, $\varepsilon_{C,0}=1$ rad s^−1^, Fig 8(p).(MP4)

S15 MovieLoose ring *ε*_U,0_ = 1 rad s^−1^, $\varepsilon_{C,0}=0.01$ rad s^−1^, Fig 8(a).(MP4)

S16 MovieLoose ring *ε*_U,0_ = 1 rad s^−1^, $\varepsilon_{C,0}=0.04$ rad s^−1^, Fig 8(b).(MP4)

S17 MovieFilament and ring patch *ε*_U,0_ = 1 rad s^−1^, $\varepsilon_{C,0}=0.16$ rad s^−1^, Fig 8(c).(MP4)

S18 MovieFilament and ring patch *ε*_U,0_ = 1 rad s^−1^, $\varepsilon_{C,0}=1$ rad s^−1^, Fig 8(d).(MP4)

S19 MovieLoose ring *ε*_U,0_ = 0.16 rad s^−1^, $\varepsilon_{C,0}=0.01$ rad s^−1^, Fig 8(e).(MP4)

S20 MovieFilament and patches *ε*_U,0_ = 0.16 rad s^−1^, $\varepsilon_{C,0}=0.04$ rad s^−1^, Fig 8(f).(MP4)

S21 MovieFilament and patches *ε*_U,0_ = 0.16 rad s^−1^, $\varepsilon_{C,0}=016$ rad s^−1^, Fig 8(g).(MP4)

S22 Movie Optimal foraging in a double gyre. Dynamics according to parameters of Table 1, Fig 10.
(MP4)

S23 MovieMinimal response to prey.Only the natural frequency ω(A) and angular diffusion D(A) change, Fig 11(a).(MP4)

S24 MovieMedium response to prey.Agents no longer avoid turbulence ($f_{C}=0.02$) and adapt ω(A) and D(A), Fig 11(b).(MP4)

S25 MovieTrapping due to prey.Responses of ω(A), D(A) and V(|U|+A), Fig 11(c).(MP4)

S26 Movie Trapping due to prey Mechanisms of S24 and S25 are combined, Fig 11(d). (MP4)

S27 Movie Ring formation *f*_U_ = 0.5 *F*_0_, Fig 14(d). (MP4)

S28 Movie Ring formation *f*_U_ = 0.025 *F*_0_, Fig 14(a). (MP4)

S29 Movie Ring formation *f*_U_ = 0.075 *F*_0_, Fig 14(b). (MP4)

S30 Movie Ring formation *f*_U_ = 0.1 *F*_0_, Fig 14(c). (MP4)
